# DCS-YOLO: Defect detection model for new energy vehicle battery current collector

**DOI:** 10.1371/journal.pone.0311269

**Published:** 2024-10-29

**Authors:** Hai Tang, Lei Yuan, Yanrong Chen, Ren Gao, Wenhuan Wu

**Affiliations:** School of Electrical and Information Engineering, Hubei Key Laboratory of Energy Storage and Power Battery, Hubei University of Automotive Technology, Shiyan, China; University of Baghdad, IRAQ

## Abstract

The future trend in global automobile development is electrification, and the current collector is an essential component of the battery in new energy vehicles. Aiming at the misjudgment and omission caused by the confusing distribution, a wide range of sizes and types, and ambiguity of target defects in current collectors, an improved target detection model DCS-YOLO (DC-SoftCBAM YOLO) based on YOLOv5 is proposed. Firstly, the detection rate of defects with different scales is improved by adding detection layers; Secondly, we use the designed DC module as the backbone network to help the model capture the global information and semantic dependencies of the target, and effectively improve the generalization ability and detection performance of the model. Finally, in the neck part, we integrate our designed Convolutional Block Attention Module (SoftPool Convolutional Block Attention Module, SoftCBAM), which can adaptively learn the importance of channels, enhance feature representation, and enable the model to better deal with target details. Experimental results show that the *mAP*_50_ of the proposed DCS-YOLO model is 92.2%, which is 5.1% higher than the baseline model. The FPS reaches 147.1, and the detection accuracy of various defect categories is improved, especially *Severely bad* and *No cover*, and the detection recall rate reaches 100%. This method has high target detection model efficiency and meets the requirements of real-time detection of battery collector defects.

## Introduction

**Fig 1:**
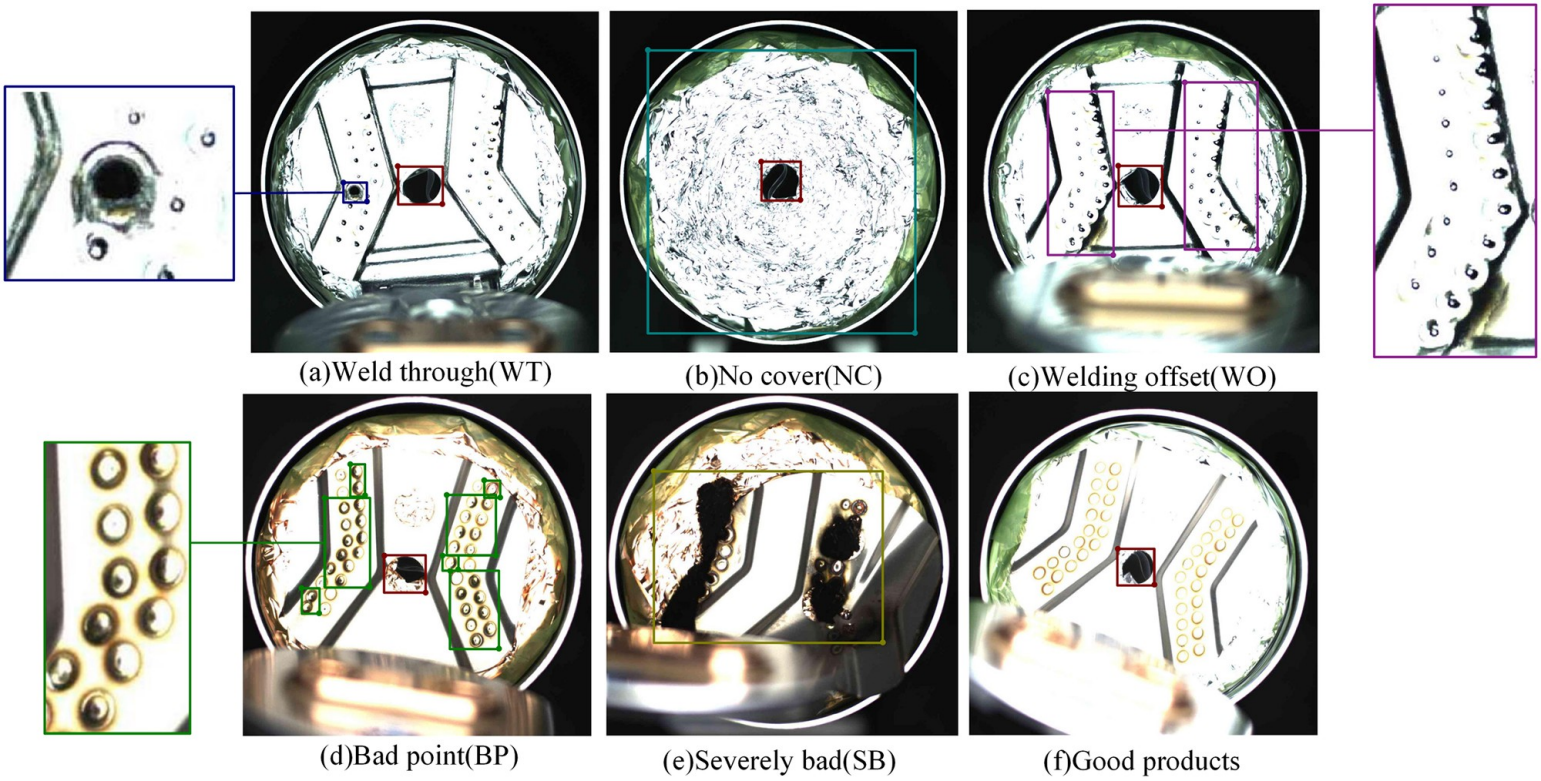
Example image of a label for BCC surface defect database.

The surge in car ownership has raised numerous pressing issues, including environmental pollution and energy crises. While meeting environmental targets and tackling these energy crises, the robust development of new energy vehicles is aptly positioned to cater to the demands of the new economic epoch. Currently, the use of automobile batteries is largely exploratory, and safety considerations are paramount. Lithium-ion batteries, a new breed of high-performance energy storage devices, possess impressive overall performance attributes, thus making them ideal for new energy passenger vehicles. Typically, all-solid-state batteries consist of a current collector, a shell, and a battery cell. Current collectors are indispensable components bridging lithium-ion batteries and external circuits [1]. One of the challenges in battery production is that the existing battery quality inspection system is not efficient enough, and prone to errors or missed inspections. In the production of all-solid-state batteries, the current collector needs to be welded separately with the battery cell and the shell, and the quality of welding quality significantly impacts the subsequent preparation of work of the all-solid-state battery. When the cell is placed in the housing, gaps between the battery cell and the shell may cause the axial line to deviate from the shell’s, leading to eccentric welding (*Welding offset*). After welding, when the battery cell expands, it may result in uneven stress at the welding spot between the cell battery cell and the shell. The current collector being placed off the axis of the battery cell will cause *Severely bad* during welding. If the welding time is too short and not firm, it will lead to the falling of current collector (*No cover*). If too long, the current collector may be damaged to different degrees (*Welding through*, *Bad point*). As shown in Fig 1, these defects affect the reliability and service life of the battery, and may lead to short circuit and explosion of the battery in severe cases, potentially causing casualties and property damage. There are few studies on the defect detection of the battery’s internal component, the current collector, thus the research in this paper is of great significance [2].

In the manufacturing process of all-solid-state batteries, surface defects in the current collector can affect the cell’s quality and functionality. These issues can be mitigated by inspecting the current collector for defects during the manufacturing process. However, manual visual inspection presents several disadvantages, including high subjectivity, a high false detection rate, low efficiency, and time-consuming manual observation. Moreover, minute defects can be easily overlooked. Therefore, there is a pressing need for efficient and comprehensive target detection algorithms in the manufacturing scenario. Various methods for detecting battery defects have been proposed and implemented in battery manufacturing. Presently, machine vision defect detection methods are primarily categorized into two types:

1. Traditional methods combine image processing with machine learning to detect features such as edges and particles of target defects. For example, edge detection algorithms like Sobel [3] and Canny [4] can identify defects in a single context. Wavelet transforms [5] and Gabor transforms [6] can detect defects with periodic textures in the background. These methods can also characterize image statistics features as grayscale differences [7] and grayscale histograms [8]. By transforming the image from the spatial domain to the frequency domain, the image can be detected. Defect classification uses traditional machine learning methods such as SVM [9] and random forest [10]. However, these traditional methods often involve manual identification and labeling of defects, which makes it difficult to identify various unknown and complex defect types in industrial scenarios due to human subjectivity. Due to the lack of detection performance, the traditional methods can not meet the actual needs of industrial application scenarios when detecting defects with complex backgrounds and irregular shapes;
2. Deep learning-based target detection algorithms. With the growing popularity of artificial intelligence, its applications are increasingly incorporated into various fields [9]. Computer vision methods, such as target detection, are now widely used to detect surface defects, saving labor costs and accelerating detection. In computer vision, convolutional neural networks (CNN) [11] have been the dominant model for vision tasks since 2012. Deep learning-based target detection algorithms can be classified into candidate area-based target detection algorithms (two-stage) and regression-based target detection algorithms (one-stage), which have significantly improved accuracy compared to traditional target detection algorithms. Typically, one-stage target detection algorithms employ an end-to-end training mode and require only one feature extraction, making them faster but less accurate and prone to missed inspections (YOLO series [12–18]). In contrast, two-stage target detection algorithms achieve higher accuracy but require more computation (R-CNN series [19–22]). Compared to the R-CNN family of algorithms, YOLO is simple and efficient, and suitable for engineering applications.

In this paper, the DCS-YOLO model is introduced to address the challenges posed by the numerous types of defects and the wide range of sizes in the battery current collector. The aim is to efficiently detect defects on the battery current collector surface. The key research contributions of this paper are as follows:

- Due to the absence of a standard defective dataset for new energy vehicle battery current collectors, a self-constructed dataset is utilized for the experiments in this paper;
- The original model is enhanced by multi-scale method, including increasing the detection scale of 10 × 10, which expands the detection range of the model. This leads to increased fusion between deep and shallow semantics, thereby reducing missed detection rates;
- The constructed DC module leverages deformable convolution (DCN) [23] and Contextual Transformer Networks (CoTNet) [24] to enhance the model’s ability to capture the relationship between the target region and its surrounding context, thereby improving the model’s capability to extract local features;
- To effectively capture regions of interest in large-sized images, an improved SoftCBAM module is introduced.

In summary, the enhanced model demonstrates superior performance across all metrics, notably improving detection accuracy for various defect categories. It significantly enhances efficiency and performance in real-time detection of defects in battery current collectors.

The structure of this article is outlined as follows: The Related Works section provides an overview of related work, discussing target detection algorithms that have been studied in the industrial domain. It primarily focuses on deep learning-based detection methods. The Proposed Methods section provides a detailed description of the specific structure of DCS-YOLO, highlighting its unique architecture and design principles. The Experimental Results and Analysis section revolves around the experimental aspects of the research. It includes creating the dataset and providing an in-depth analysis of the conducted experiments. The Conclusion section summarizes the main findings and contributions of this article. Potential future research directions and further development areas are also proposed.

## Related works

This section presents target detection algorithms studied in the industrial domain, focusing on deep learning methods.

Hu *et al* [25] proposed an effective model for detecting defects in lithium battery steel casings. The proposed model demonstrates superior overall performance with an average precision of 88.3%, which is 6.9% higher than the YOLOv5s model. This lays a foundation for the industrial implementation of real-time detection in lithium battery production. Badmos *et al* [26] proposed a novel method for detecting microstructural defects in lithium-ion battery electrodes using convolutional neural networks. The detection results were significantly better than those achieved by traditional machine learning models and even outperformed CNN models trained directly on battery data. Wu *et al* [27] proposed a cross-domain FSL (few-shot learning) method for defect classification of lithium-ion batteries with an improved Siamese network called BSR-SNet. The method achieved an mAP value of 93.3%, making it applicable to the classification of aluminum/steel shell lithium batteries. Li *et al* [28] propose an ensemble framework for industrialized rail defect detection. On they collected dataset with 8 defect classes, their algorithm achieves a 7.4% higher mAP.5 compared to YOLOv5 and a 2.8% higher mAP.5 compared to Faster R-CNN. Zhang *et al* [29] propose a lightweight YOLOv7 insulator defect detection algorithm for UAV-based transmission line insulator inspection. With a single image detection time of 105ms and a capture rate of 13FPS on the Jetson Nano, the improved model meets the demands of real-time detection. Wang *et al* [30] have proposed applying deep learning based object detection technology to surface defect detection of electronic panels to address the challenges of extreme irregularity and small target characteristics. The proposed method demonstrating an increase of 13.506 percentage points over YOLOV5 and 33.457 percentage points higher than Retinanet in map_0.5 metric on the electronic panel defect dataset. Xu *et al* [31] propose a deep learning defect detection method based on an enhanced YOLOv5 algorithm, aimed at addressing the low efficiency of manual detection in metal surface defect detection. The algorithm demonstrates significant improvements in mAP and FPS on the dataset, enabling quick and accurate identification of metal surface defects with practical industrial applications.

The studies mentioned above on defect detection have yielded impressive results within their respective application domains. However, several challenges persist in practical applications:

- Some studies focus on detection targets with fixed sizes and features. This approach, however, lacks generalizability due to the wide size range and diverse features of battery current collector defects;
- Certain studies employ traditional detection methods, which exhibit poor robustness. Certain utilize two-stage target detection algorithms, which has the problems of slow model training, low detection, and high false detection rates. This makes it challenging to balance detection accuracy and model complexity;
- Some studies have adopted simpler models and rely on outdated improvement methods. Consequently, the detection accuracy is compromised, making it hard to meet application requirements.

Usually, these methods aim to enhance detection accuracy but at the cost of real-time performance. Alternatively, they may improve real-time performance yet not sufficiently enhance detection accuracy. Therefore, we propose DCS-YOLO for defect detection in battery current collectors. This research aims to improve the detection accuracy of the five types of defects in the current collectors while maintaining low parameter and computational requirements.

## Proposed methods

**Fig 2:**
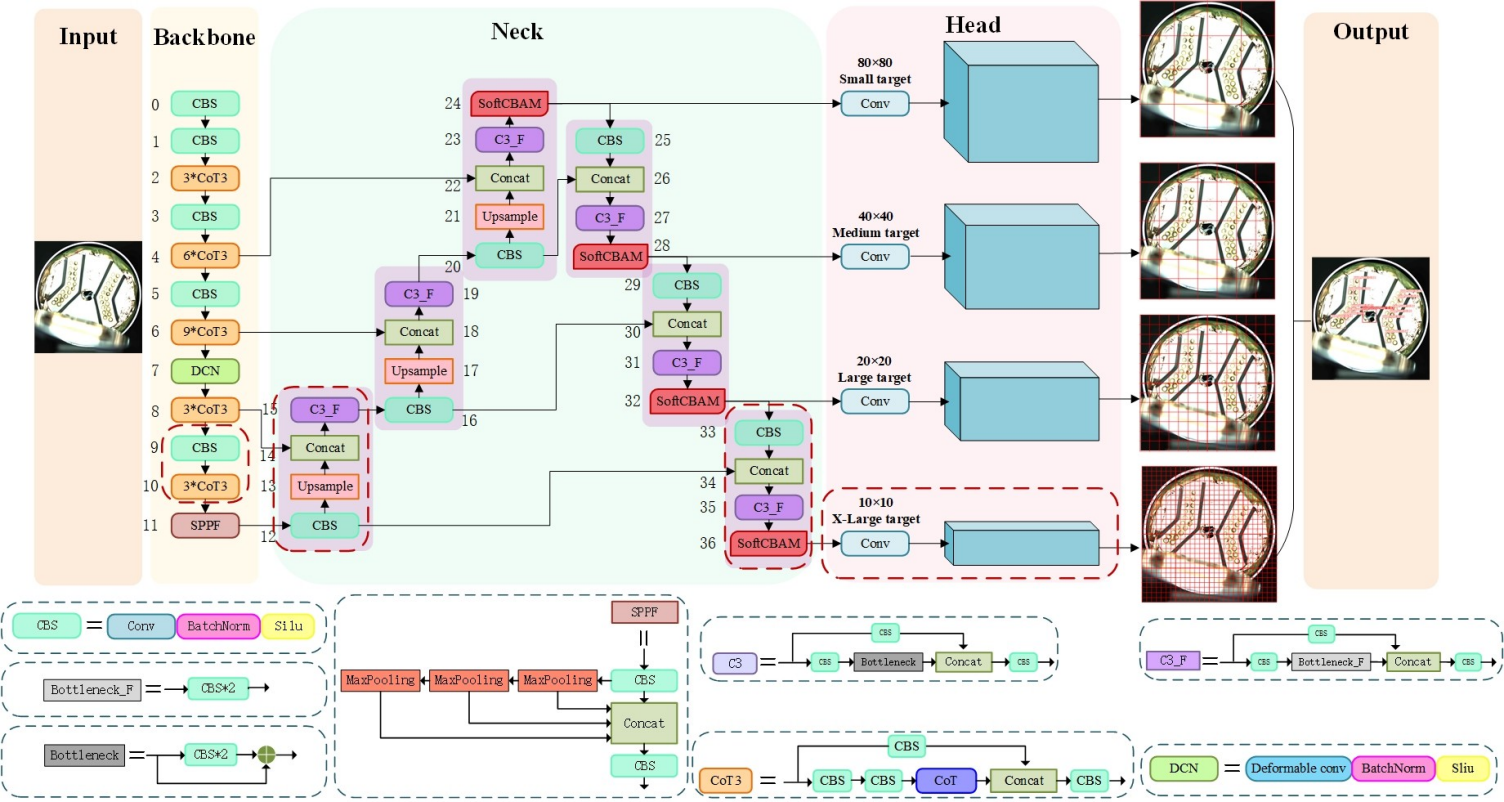
Overall architecture of the DCS-YOLO.

To enhance the performance of deep learning-based defect detection models for new energy vehicle battery current collectors, this paper designs inspiration from existing literature and designs a defect detection model based on deformable convolution and attention mechanisms: DCS-YOLO. The YOLOv5 network serves as the benchmark model for defect detection, and the improved model is illustrated in Fig 2.

### New detection layer

Due to the large original sample size of the current collector in new energy vehicle batteries and the wide range of sizes and types of surface defects, the original YOLOv5 model struggles to accurately detect Severely bad, which are often oversized targets that cannot be fully covered with a 20 × 20 scale. This limitation results in lower detection accuracy for Severely bad categories. To obtain features of finer granularity and more detailed location information, a 10 × 10 feature scale was added to focus on these ultra-large objects. The feature map 10 × 10 was used with the twice-up sampled feature map 20 × 20 to fusion to detect ultra-large-scale targets. The new detection layer is well-suited to the defect category of Severely bad, making the output layer after fusion mapping richer in features and enhancing the network’s expressiveness for both shallow and deep semantic information. The improved areas are shown in the red dashed box in Fig 2.

### DC module

Target defects in current collector objects exhibit scattered distribution and blurry characteristics. In order to better capture the details and shape information of the targets and make better use of the contextual information around the targets to improve the model’s performance and robustness, the DC module is proposed as the backbone network. The DC module is composed of the DCN and CoT3 modules.

#### Deformable convolution

**Fig 3:**
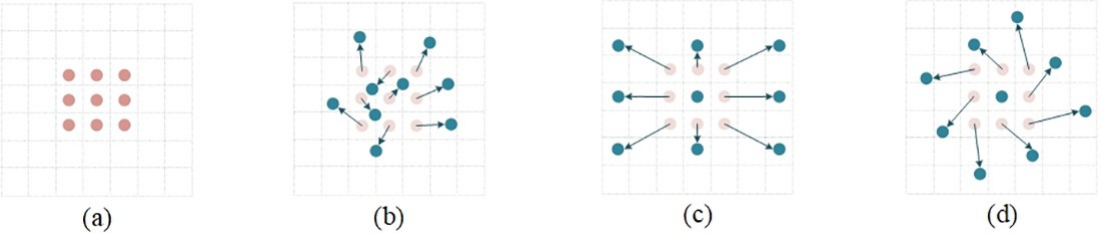
Schematic diagram of sampling position of traditional convolution and deformable convolution. (a) traditional convolution. (b)(c)(d) Deformable convolution.

**Fig 4:**
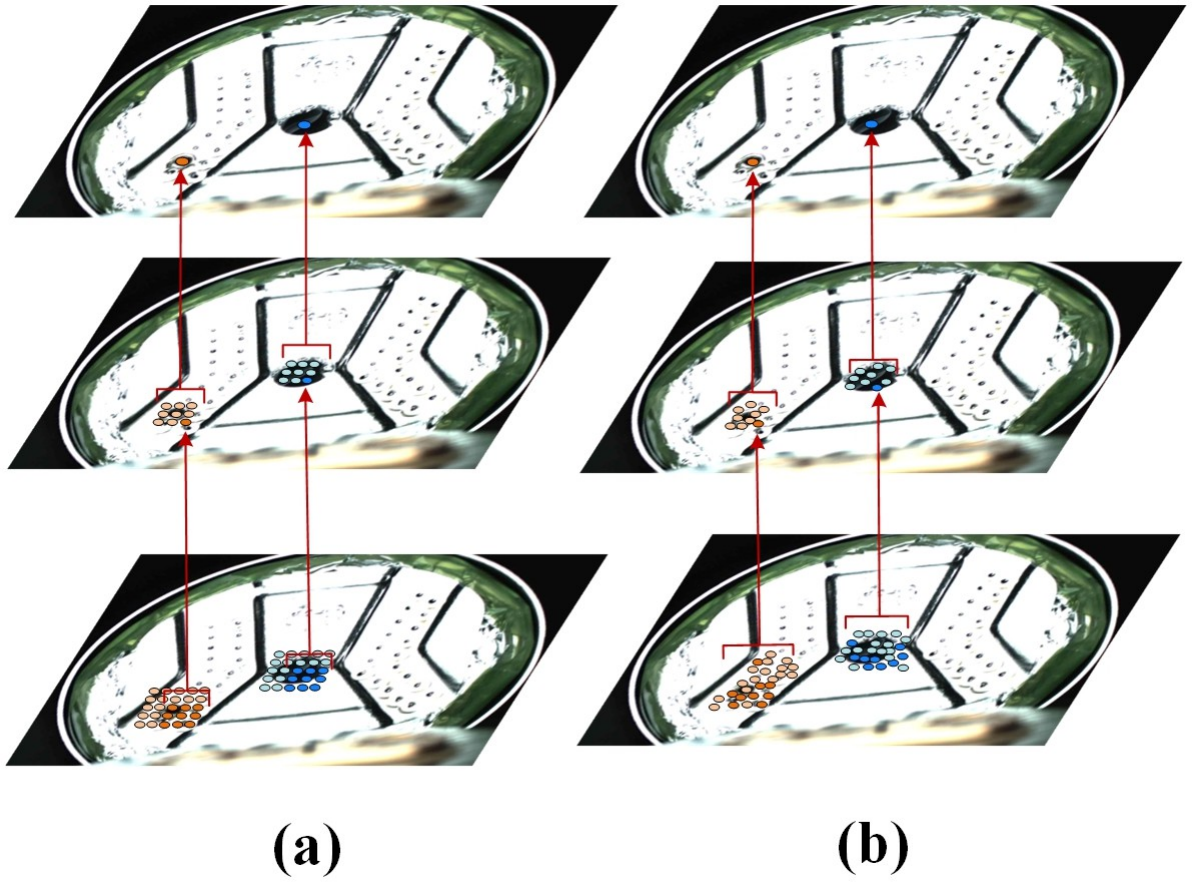
The feature extraction process of traditional convolutions and deformable convolutions. (a) traditional convolution. (b) Deformable convolution.

**Fig 5:**
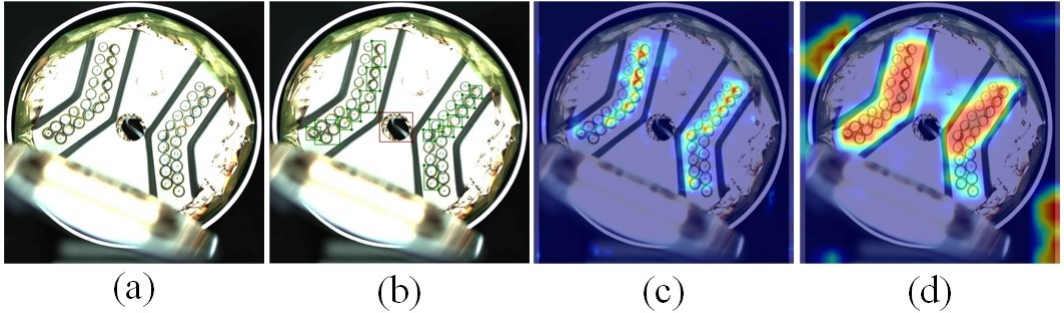
Comparison of traditional convolution and deformable convolution heat map. (a) Original image. (b) Label image. (c) traditional Convolution. (d) Deformable Convolution.

Convolutional layers are used to extract feature information from images. Fig 3 illustrates the sampling positions. As depicted in (b), each convolution kernel of deformable convolution has a learnable parameter biases, allowing the sampling points of deformable convolution to adjust based on feature maps adaptively. Regarding the target defects on the current collector, the feature extraction processes of traditional and deformable convolution are illustrated in Fig 4. The heatmaps generated by traditional convolution and deformable convolution are shown in Fig 5. The information extracted by deformable convolution is more comprehensive, and the adaptability of the network to the features with significant shape variations is enhanced.

The computation process of deformable convolution improves upon traditional convolution. It can be divided into two steps: The feature map *x* is sampled using a regular mesh *R*, and the sum of each sampled point is multiplied with its corresponding weight. The traditional convolutional structure can be defined by Formula (3).

$$\begin{array}{c} y(p_{0})=\sum_{p_{n}\in R}w(p_{n})\cdot x(p_{0}+p_{n}), \end{array}$$ (1)

*p*_*n*_ represents the enumeration value for each position in the grid *R*, and *w*(*p*_*n*_) denotes the weight corresponding to that point. Deformable convolution expands the regular grid *R* by incorporating offset values {Δ*p*_*n*_|*n* = 1⋯*N*}, *N* = |*R*|. The formula for deformable convolution is shown as Formula (4).

$$\begin{array}{c} y(p_{0})=\sum_{p_{n}\in R}w(p_{n})\cdot x(p_{0}+p_{n}+\Delta p_{n}), \end{array}$$ (2)

Since Δ*P*_*n*_ typically takes decimal values, bilinear interpolation calculates the transformed feature values. The following formula can express it:

$$\begin{array}{c} x(p)=\sum_{q}g(q_{x}\cdot p_{x})\cdot g(q_{y}\cdot p_{y})\cdot x(q), \end{array}$$ (3)

*p* = *p*_0_ + *p*_*n*_ + Δ*p*_*n*_, *q* represents the enumeration of *x* spatial positions in the feature map. *g*(*q*_*x*_ ⋅ *p*_*x*_) and *g*(*q*_*y*_ ⋅ *p*_*y*_) are bilinear interpolation functions.

After extracting effective features from the input image through the Backbone section, the Deformable Convolution module further extracts features with positional offsets. The obtained features are then subjected to Batch Normalization and activated using the SiLU activation function. The process above is completed by the DCN module, as shown in Fig 2. The DCN module is introduced in the newly added 20 × 20 detection scale block within the Backbone to expand the receptive field, enrich the extraction of target features, and enhance the detection capability of irregular objects. The improved positioning is indicated in Fig 2.

#### CoT3 module

**Fig 6:**
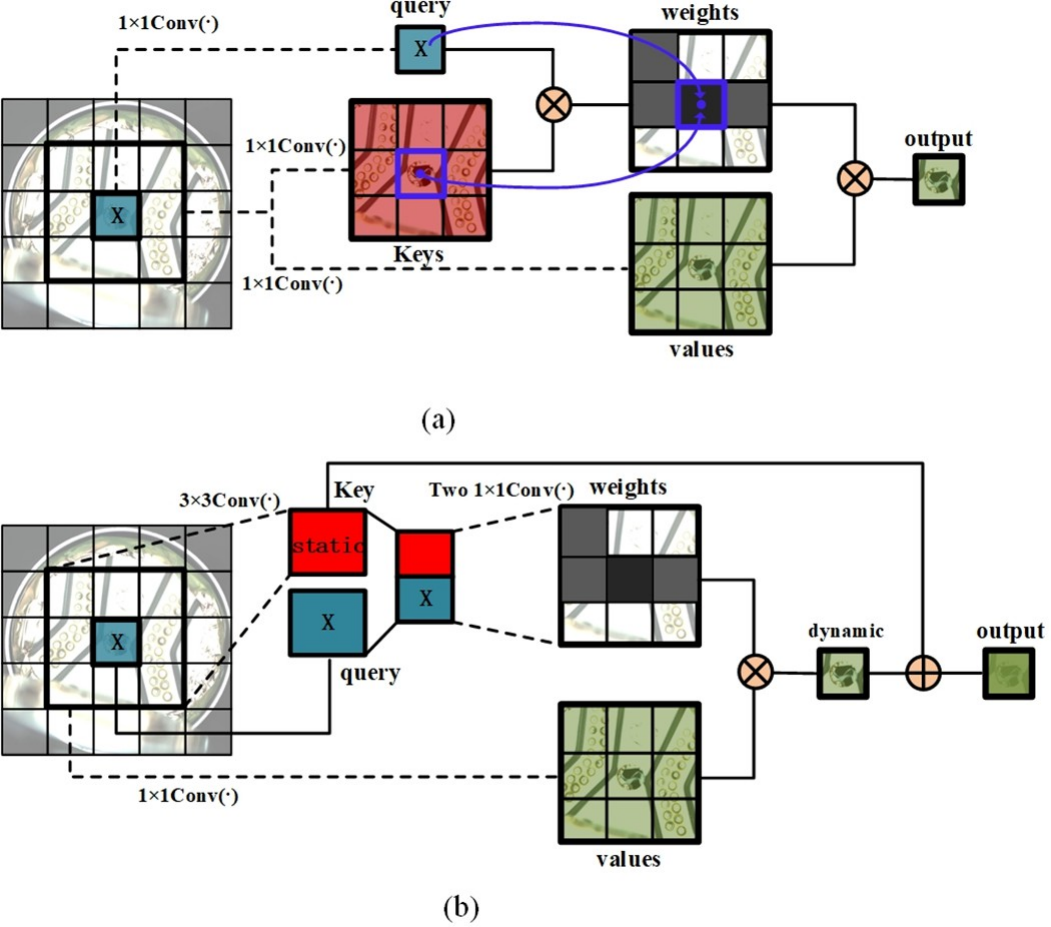
Comparison between conventional self-attention and Contextual Transformer (CoT) block. (a) Conventional selfattention block. (b) Contextual Transformer (CoT) block.

We introduce a Contextual Transformer (CoT) module into the backbone network to further enhance the ability to gather global contextual information. Fig 6 compares the traditional self-attention mechanism and our CoT module. The traditional self-attention module only exchanges information within the spatial domain, overlooking the rich contextual information between neighboring keys.

**Fig 7:**
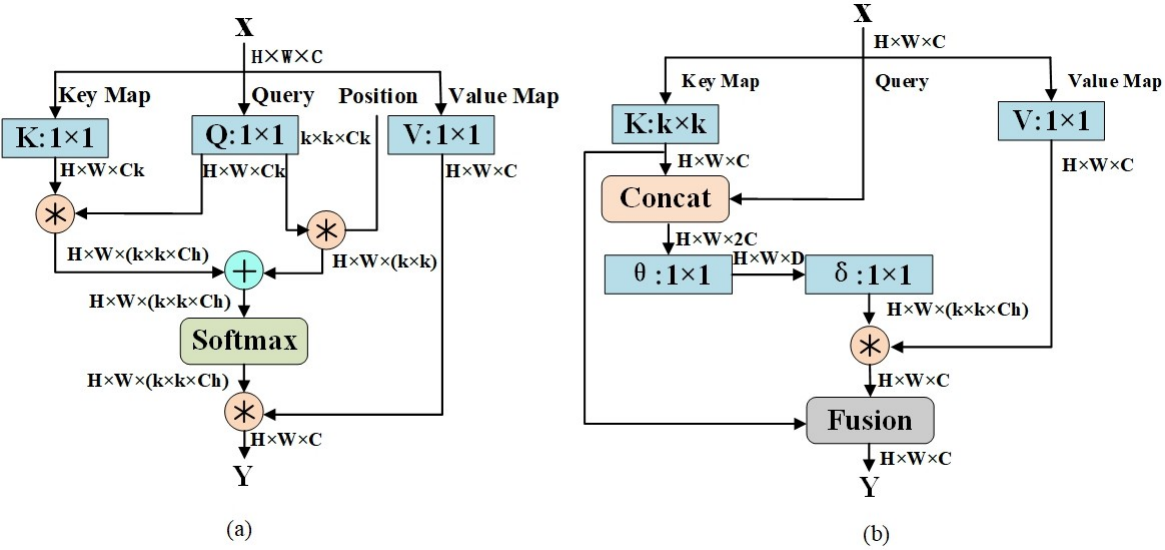
(a) Conventional self-attention block. (b) Contextual Transformer (CoT) block.

The principle of the traditional attention mechanism is illustrated in Fig 7(a). Given a 2D feature map of size, *H* × *W* × *C*, where *H* corresponds to height, *W* corresponds to width, and *C* corresponds to the number of channels. By applying *Q* = *XW*_*q*_, *K* = *XW*_*k*_ and *V* = *XW*_*v*_, we obtain keys, queries, and values. Here, the (*W*_*q*_, *W*_*k*_, *W*_*v*_) denotes the embedding matrix, and each embedding matrix is implemented using a 1 × 1 convolution. Subsequently, we can obtain the local relationship matrix $R(R\in R^{H\times W(k\times k\times C_{h})})$ between *K* and *Q*:

$$\begin{array}{c} R=K⊗Q, \end{array}$$ (4)

*C*_*h*_ is the number of heads, and ⊗ denotes the element-wise matrix multiplication.

To enrich the local relationship matrix *R* by adding positional information. The expression is as follows:

$$\begin{array}{c} \hat{R}=R+P⊗Q, \end{array}$$ (5)

*P* denotes the relative positional information. We utilize softmax to obtain the attention matrix $A=Softmax(\hat{R})$, then learn the local attention matrix based on *V*. The final output is as follows:

$$\begin{array}{c} Y=V⊗A, \end{array}$$ (6)

The principle of the CoT module is shown in Fig 7(b).

Given a 2D feature map $X(X\in R^{H\times W\times C})$, the keys, queries, and values are denoted as *K* = *X*, *Q* = *X* and *V* = *XW*_*v*_, respectively. In contrast to the traditional self-attention mechanism that applies a 1 × 1 convolution to the keys, the CoT module utilizes a *k* × *k* group convolution to extract contextual information. The resulting *K*^1^ reflects the contextual information between adjacent key values and serves as a static contextual representation of the input *X*. Next, *K*^1^ is concatenated with *Q*, and the attention matrix is obtained through two consecutive 1 × 1 convolution operations:

$$\begin{array}{c} A=[K^{1},Q]W_{\theta }W_{\delta }, \end{array}$$ (7)

The feature map *K*^2^ is obtained by aggregating all the values *V*. The expression is as follows:

$$\begin{array}{c} K^{2}=V⊗A, \end{array}$$ (8)

As the feature map *K*^2^ captures dynamic feature interactions, it is referred to as the dynamic contextual representation of the input. Therefore, the output *Y* of the CoT module is a fusion of the static contextual representation *K*^1^ and the dynamic contextual representation *K*^2^ achieved through attention mechanisms.

Consequently, to capture contextual information between adjacent keys, improvements are made based on the C3 module by integrating the CoT module into the backbone feature extraction network. As shown in Fig 2, after replacing the CBS module with the CoT module, the C3 structure is reconfigured to form a new CoT3 module. This allows the network model to acquire global information and improve the detection performance of defects in the battery current collector but also applies the new CoT3 module to low-resolution feature maps, reducing expensive computation and storage costs.

In summary, our proposed DC module improves the detection capability of the model. On the one hand, by introducing the DCN, the sampling positions of the convolutional kernel can be adaptively adjusted to model the shape variations of the targets. This enables the model to better adapt to different targets’ shape and scale changes. On the other hand, by introducing the Contextual Transformer, the surrounding context information is effectively integrated into each grid by learning the importance of context information. This helps the model better understand and localize the targets.

### SoftCBAM module

In order to effectively enhance the model’s ability to extract features of battery current collector defects, SoftPool is introduced to retain more information in the downsampling activation mapping and SoftCBAM is designed. SoftCBAM is incorporated into the Neck section of YOLOv5. The improved location is shown in Fig 2.

#### The principle of SoftCBAM module

**Fig 8:**
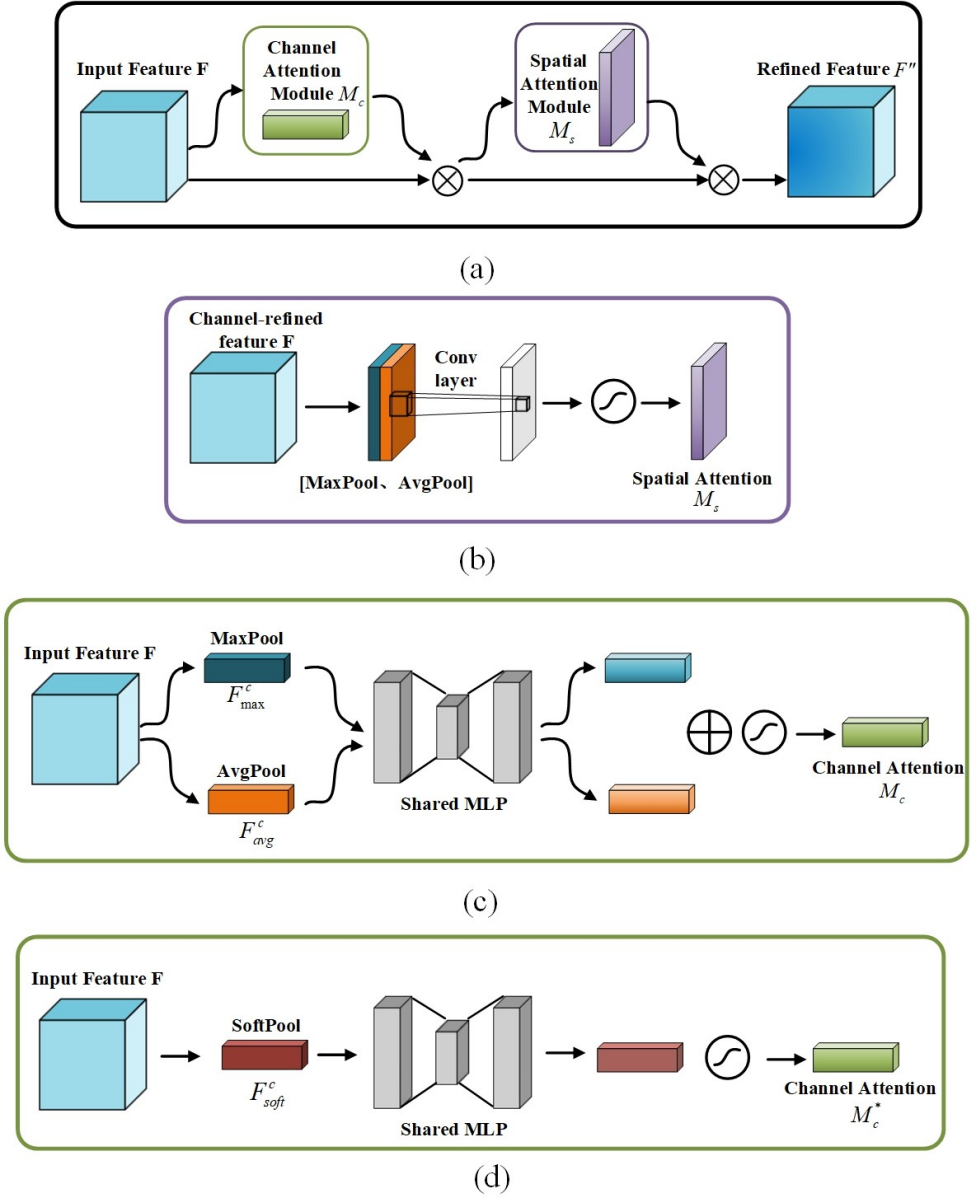
Comparison of CBAM and SoftCBAM. (a) CBAM. (b) Spatial Attention Module. (c) CBAM Channel Attention Module. (d) SoftCBAM Channel Attention Module.

1. *CBAM:*CBAM [32] is a hybrid attention module combining channel and spatial attention. This combination helps CBAM to achieve better results in improving model accuracy and suppressing irrelevant noise information.
  As shown in Fig 8(a), given a 2D feature map of size *H* × *W* × *C*, where H represents the height, *W* represents the width, and *C* represents the number of channels in the feature map. The CBAM block sequentially generates attention maps through two dimensions, followed by element-wise multiplication between the input and attention maps for adaptive feature optimization. The output of the convolutional layer is weighted by the channel attention module first and then by the spatial attention module, resulting in the final weighted output. The entire process can be formulated as follows:
  $$\begin{array}{c} F^{\prime }=M_{c}(F)⊗F,F^{\prime \prime }=M_{s}(F^{\prime })⊗F^{\prime }, \end{array}$$ (9)
  ⊗ denotes element multiplication, $M_{c}\in R^{C\times 1\times 1}$ denotes channel attention, and $M_{s}\in R^{1\times H\times W}$ denotes spatial attention.
2. *SoftCBAM channel attention module:* The CBAM module computes attention maps in two dimensions by extracting input attention information through max pooling and average pooling. However, for battery current collector defects with multiple categories, uncertain edges, and high background overlap, the CBAM module may lead to the loss of detailed information on the battery current collector, and insufficient detection performance of the model. Therefore, in this paper, SoftCBAM is designed with SoftPool to maximize the preservation of detailed information and enhance the extraction of important features.
  Currently, the commonly used pooling operations (average pooling and max pooling) tend to lose the majority of information in the image during the pooling process, thus leading to a decrease in the overall network performance. SoftPool [33], on the other hand, uses softmax weighting to preserve the fundamental properties of the input while amplifying stronger feature activations, thus minimizing information loss caused by pooling while retaining its functionality.
  SoftPool utilizes the natural exponential function with base *e* to ensure that larger activation values have a greater impact on the output. Additionally, due to its differentiability, every activation within the local neighborhood *R* is assigned at least a minimal gradient value during the backpropagation phase. In the SoftPool pooling process, each activation *a*_*i*_ is assigned a weight *w*_*i*_. This weight is the ratio of the natural exponentiation of the activation relative to the sum of the natural exponentiations of all activations within the neighborhood *R*:
  $$\begin{array}{c} w_{i}=e^{a_{i}}(\sum_{j\in R}e^{a_{j}}{)}^{-1}, \end{array}$$ (10)
  The SoftPool output value is obtained by taking the weighted sum of all activations within the kernel neighborhood:
  $$\begin{array}{c} \bar{a}=\sum_{i\in R}w_{i}a_{i}, \end{array}$$ (11)
  Fig 8(d) shows the structure of the SoftCBAM channel attention module. Considering the limitations of exponential calculations in the SoftPool pooling process on model efficiency, this paper only uses SoftPool in the channel dimension of CBAM to replace max pooling and average pooling. While preserving more detailed defect features, it enhances the extraction of significant defect features. Firstly, the input features $F\in R^{C\times H\times W}$ are compressed in the spatial dimension through SoftPool operation to obtain spatial background feature representation $F_{soft}^{c}\in R^{C\times 1\times 1}$, which is then fed into an MLP. The MLP output features undergo sigmoid activation to generate the input features:
  $$\begin{array}{c} M_{c}^{*}(F)=\sigma (MLP(SoftPool(F)))=\sigma \left(W_{1}\left(W_{0}\left(F_{soft}^{c}\right)\right)\right), \end{array}$$ (12)
  Next, the element-wise multiplication is performed between $M_{c}^{*}$ and the input feature *F*, resulting in the input feature required for the spatial attention module:
  $$\begin{array}{c} F^{\prime }=M_{c}^{*}\left(F\right)⊗F, \end{array}$$ (13)

In summary, the model’s detection range have been expanded by adding a 10x10 detection layer, allowing for the detection of features from battery current collector of different sizes. The fusion of deep and shallow semantics have also been improved, resulting in a reduction in the false-negative rate. By incorporating the proposed DC module as the backbone network, we can better identify complete features on the surface of defects, thereby ensuring local feature extraction capability and improving the model’s ability to capture global information while reducing the parameter count. Additionally, by integrating the designed SoftCBAM module into the feature fusion network, we reduce information loss and enhance the model’s recognition capabilities. The entire process is shown in Fig 2.

## Experimental results and analysis

### BCC surface defect database

**Fig 9:**
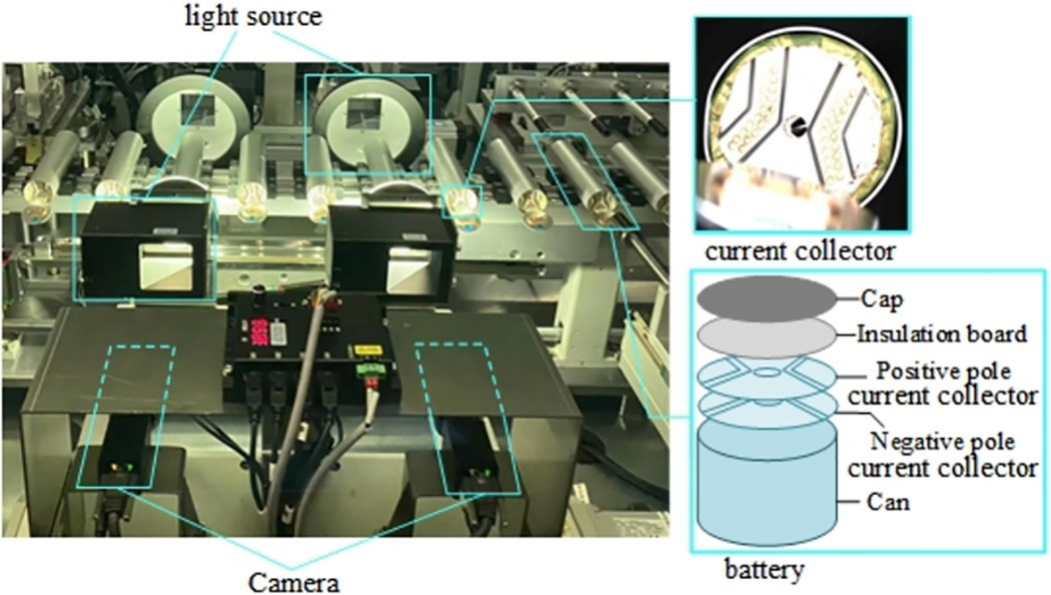
Image acquisition system for battery current collector.

The BCC surface defect database used in this study was self-constructed, utilizing real-world data of defects in lithium iron phosphate automotive battery current collectors collected from detection equipment on a dust-free constant temperature production line. A conveyor belt transported the batteries to the detection equipment and illuminated them with using a white coaxial light source. The current collector images were captured by a Basler camera model acA2500-14gc, equipped with a 25mm lens, model SC-DLH25-5M. The CPU model of the data acquisition computer was I7-7700, running on the Windows 10 Professional operating system. Fig 9 shows an example of the on-site image capture in the automated production line. The Lableme data annotation tool is employed to label the positions and categories of all image defects. The annotation results were repeatedly verified and corrected to avoid any subjective influence on experimental results. Fig 1 represents a typical image from the BCC dataset, and five common defect types were defined: *Weld through(WT)*, *Welding offset(WO)*, *No cover(NC)*, *Bad point(BP)*, and *Severely bad(SB)*.

Due to the similarity between defects like the *Weld through* of battery current collector in new energy vehicles and central hole features, the central holes were labeled as positioning holes to prevent model misidentification. To balance the data for five types of defects and avoid adverse effects, a small number of unclear images were excluded during the labeling process. In the end, a dataset of 2000 defect images was obtained. During the training, the dataset was randomly divided into 1400 training samples, 180 validation samples, and 420 testing samples, in a ratio of 7:1:2. The number of samples for each defect is shown in Table 1.

**Table 1:** Number of samples per defect.

	PH	BP	WO	NC	SB	WT
Number of samples	1890	1470	479	327	103	505

The ratio of training, validation, and testing sets is an important parameter that affects the detection performance of the model during the training process of the current collector data. In order to ensure the rigor of the experimental results, this study compared three commonly used data set division rules for industrial defect data training: 6:2:2, 8:1:1, and 7:1:2.

The defect detection abilities of the original and improved models were evaluated on two types of data sets. The experimental results are shown in Table 2. Overall, the optimized model performed well under all three different scenarios but achieved the best performance when the 7:1:2 ratio was used, which fully demonstrated its potential. Therefore, this study adopted the 7:1:2 ratio as the basis for data set division in both ablation experiments and comparative experiments.

**Table 2:** Experiments with different data set ratios.

Ratio	Model	P	R	*mAP*_50_(%)
8:1:1	Baseline	87.6	85.4	87.0
	DCS-YOLO	90.4	88.3	91.5
6:2:2	Baseline	88.3	82.8	86.6
	DCS-YOLO	88.8	89.9	90.6
7:1:2	Baseline	87.3	85.6	87.1
	DCS-YOLO	90.7	90.2	92.2

### Experimental environment and parameter settings

The experimental environment and parameter settings are shown in Table 3.

**Table 3:** Experimental environment and parameter configuration.

Projects	Content	Hyper-Parameters	Value
CPU	Intel(R)Core(TM) i5-12600KF	Batch-size	16
RAM	16G	Epoch	300
GPU	NVIDIA RTX3080×2	Image Size	640
CUDA	Cuda11.3 and Cudnn10.1	Initial Learning Rate	0.01
Deep Learning Framework	Pytorch 1.12.1	Momentum	0.937
Operating System	Ubuntu 18.04.6	Weight Decay Coefficient	0.0005
Programming Languages	Python 3.9	Patience	100
Initialization Method	Pretrained Model Initialization	Confidence Threshold	0.25
Optimization Method	Adam	IoU Threshold for NMS	0.45

### Evaluation indicators

Detection accuracy and computational cost are the main criteria for evaluating algorithm performance. We use *precision* (*P*), *recall* (*R*), *average precision* (*AP*), and *mean average precision* (*mAP*) as metrics for detection accuracy, and *GFLOPs* and *frames per second* (*FPS*) as indicators for computational cost. The calculation formulas are as follows:

$$\begin{array}{c} AP=\int_{0}^{1}pdR, \end{array}$$ (14)

$$\begin{array}{c} mAP=\frac{\sum_{i=1}^{N}Ap_{i}}{N}, \end{array}$$ (15)

$$\begin{array}{c} Recall=\frac{TP}{TP+FN}, \end{array}$$ (16)

$$\begin{array}{c} Precision=\frac{TP}{TP+FP}, \end{array}$$ (17)

$$\begin{array}{c} GFLOPs=\frac{Floating\;Point\;Operations\;Per\;Second}{\left(Run\;time\;in\;seconds\right)\times 10^{9}}. \end{array}$$ (18)

In the equations above, *TP* represents true positives, *FP* represents false positives, and *FN* represents false negatives. *AP* is obtained through the integration of *precision* (*P*) and *recall* (*R*). The *Precision-Recall* curve can illustrate the algorithm’s overall performance. A higher *AP* value indicates better detection performance of the model. The *mAP* value is the average of the *AP* values for the six detection targets. A higher *mAP* value corresponds to better detection performance of the model and higher recognition accuracy.

### Experimental analysis of improved DCN

Based on YOLOv5, an improved multi-scale experiment called YOLOv5-A was conducted. In order to verify the effectiveness of incorporating DCN and control the model’s parameter count, we fused DCN in four feature dimensions of the backbone, labeled as DCN_S, DCN_M, DCN_L, and DCN_X. The symbols S (Small), M (Medium), L (Large), and X (X-Large) represent four detection scales of 80 × 80, 40 × 40, 20 × 20, and 10 × 10, respectively, corresponding to Fig 2. Other parts remained unchanged. Comparative experiments with YOLOv5 were carried out on the BCC surface defect database, and the experimental results are shown in Table 4. The “✓” indicates the use of a certain improvement method.

**Table 4:** Verification experiment of fusing deformable convolutions.

Models	S	M	L	X	Weights(M)	GFLOPs	P	R	*mAP*_50_(%)
YOLOv5					14.5	15.8	87.3	85.6	87.1
YOLOv5-A-DCN_S	✓				25.5	16.5	88.2	89.3	90.4
YOLOv5-A-DCN_M		✓			26.5	16.3	89.4	86.5	89.5
YOLOv5-A-DCN_L			✓		28.0	16.2	90.5	88.9	91.0
YOLOv5-A-DCN_X				✓	30.1	16.2	90.1	87.2	88.7
YOLOv5-A-DCN_SL	✓		✓		28.2	16.8	89.2	89.8	90.4
YOLOv5-A-DCN_SMLX	✓	✓	✓	✓	25.5	16.5	91.7	85.8	89.8

According to Table 4, after introducing DCN, the *mAP*_50_ of DCN_S, DCN_M, DCN_L, and DCN_X increased by 3.3%, 2.4%, 3.9%, and 1.6% respectively, compared with the baseline model. The fusion of multiple deformable convolutions did not achieve better results. Therefore, fusing DCN at the 20 × 20 detection layer yielded the best performance. The experimental results indicate that the integration of DCN has enlarged the receptive field of the feature maps to a certain extent, improving the accuracy and robustness of object detection.

### Ablation experiment

DCS-YOLO, an improved model based on YOLOv5 was developed. The improvements include the addition of a 10 × 10 feature scale, a DC module, and SoftCBAM. To thoroughly verify the effectiveness of proposed improvements, ablation experiments were conducted on the BCC surface defect database. Each improvement was sequentially embedded into the baseline model, and the same training parameters and environmental conditions were used for each experiment. The experimental results are shown in Table 5.

**Table 5:** Ablation experiment.

4scal	DC module		SoftCBAM	Weights(M)	GFLOPs	*mAP*_50_(%)
	DCN	CoT3				
				14.5	15.8	87.1
✓				25.2	16.2	90.3
	✓			19.4	15.9	87.4
		✓		14.5	15.8	87.8
	✓	✓		19.4	15.9	88.6
			✓	14.5	18.9	87.6
✓	✓	✓		28.0	16.3	91.3
✓			✓	25.3	16.2	90.7
	✓	✓	✓	19.5	16.0	87.8
✓	✓	✓	✓	28.1	16.2	92.2

Table 5 demonstrates that implementing the 4scale enhancement alone increases *mAP*_50_ by 3.2% compared to the baseline network. This indicates that integrating a 10 × 10 detection scale enables the model to better adapt to objects of various sizes, effectively enhancing detection accuracy. When applying the DCN module or CoT module individually, *mAP*_50_ improves by 0.3% and 0.7% respectively over the baseline network. However, their combination results in a 1.5% increase in *mAP*_50_ compared to the baseline, highlighting how DC enhances the model’s discriminative ability by incorporating contextual information and improving detection performance. When SoftCBAM is applied alone, its adaptation to image variations improves *mAP*_50_ by only 0.5% compared to the baseline, indicating its effectiveness in enhancing model adaptability despite the backbone network’s limited feature extraction capabilities. Pairwise combinations of 4scale, DC, and SoftCBAM show varying degrees of improvement in *mAP*_50_ over the baseline network. Yet, combining all three enhancements achieves the highest *mAP*_50_ of 92.2%, a 5.1% increase over the baseline model. Despite slight increases in parameters and GFLOPs, these remain within acceptable limits. The modules and methods in this study notably enhance network performance to a certain extent. However, combining modules proves more beneficial than summing individual improvements.

### Comparative experiment

#### Comparative experiment of different attention models and SoftCBAM

To validate the effectiveness of our proposed SoftCBAM, several comparative experiments were conducted by introducing other attention modules at the same location. The results are shown in Table 6. From Table 6. It can be observed that the other attention models demonstrated some improvement in *mAP*_50_. Nevertheless, our proposed SoftCBAM achieved the highest increase in mAP, reaching 92.2%. Table 7 shows the F1 scores for each defect, all of which are maximized when using SoftCBAM.

**Table 6:** Comparative experiments on different attention mechanisms.

Models	GFLOPs	P	R	*mAP*_50_(%)
ECA-Net [34]	16.3	86.5	85.4	90.4
ESENet [35]	16.3	88.2	87.1	90.8
SENet [36]	16.3	89.0	87.3	88.7
GAM [37]	20.3	87.7	86.8	88.9
CBAM	16.3	90.4	90.2	91.8
SoftCBAM	16.2	90.5	90.6	92.2

**Table 7:** F1 values of different attention mechanisms on various defects.

Models	PH	BP	WO	NC	SB	WT	ALL
ECA-Net	98.9	68.4	94.0	90.6	87.2	71.3	85.0
ESENet	98.6	69.3	95.3	91.7	95.2	71.8	87.0
SENet	98.9	68.6	95.5	98.0	92.1	75.0	88.0
GAM	98.9	66.4	95.2	97.6	91.8	73.0	87.0
CBAM	98.8	70.1	95.4	98.0	99.0	74.0	89.0
SoftCBAM	98.9	71.0	95.5	98.3	99.5	78.0	90.0

**Fig 10:**
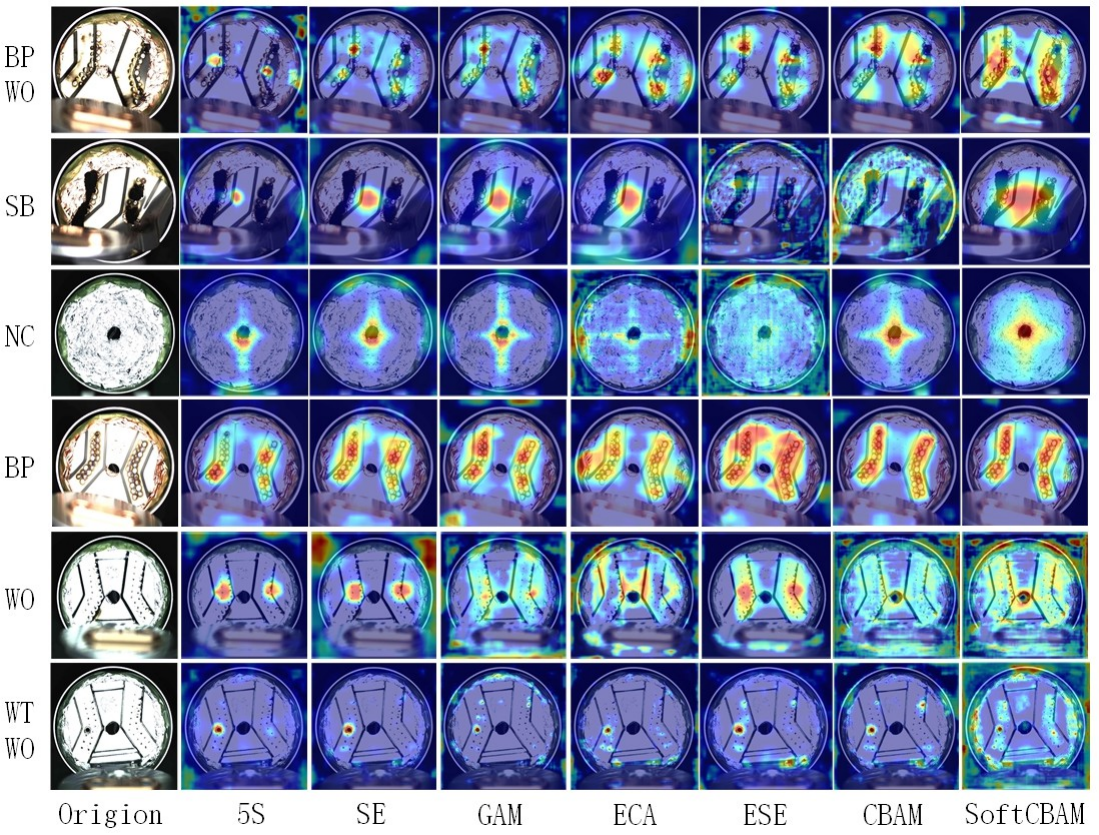
Experimental results of heat map of six attention mechanism.

The heatmap in Fig 10 is generated by six corresponding attention mechanisms on five types of defect samples. The heatmap visually displays the attention regions of each attention mechanism module, with color intensity indicating the strength of attention. The analysis is conducted from top to bottom.

When dealing with defects of the *Bad point* and *Welding offset* types, the attention regions of other attention mechanisms are too small, and only SoftCBAM pays sufficient attention to the complete feature information. When dealing with *Severely bad* and *No cover* types, the features are concentrated in the center of the image, with SoftCBAM paying attention to the complete feature information. In contrast, other attention mechanisms have lower attention to background areas. When dealing with the *Bad point* type of defect, the baseline model has a small attention region and cannot extract complete feature information. SE (Squeeze-and-Excitation), GAM (Global Attention Mechanism), ECA (Efficient Channel Attention), and CBAM (Convolutional Block Attention Module) ignore some small targets due to noise interference, leading to varying degrees of missed or false detection. ESE (Excitation and Spatial Encoding) pays attention to useless areas. SoftCBAM, on the other hand, has a high attention intensity in the bad point region, which can eliminate noise or redundant information in the input data. The color intensity changes smoothly, presenting a balanced attention distribution across different positions. When dealing with the *Welding offset* type of defect, CBAM has weak color intensity and poor attention capability. Other attention mechanisms excessively focus on useless areas, failing to capture the local welding offset features. However, SoftCBAM pays sufficient attention to the position information and concentrates high-intensity colors on the target area of the welding offset. When dealing with defects of the *Weld through* and *Welding offset* types, all attention mechanisms perform well for solder bridges but ignore the local welding offset information. Only SoftCBAM has a higher attention level in the welding offset area.

#### Comparative experiments of different models on BCC surface defect database

To demonstrate the superior performance of the proposed method, DCS-YOLO was compared with multiple classical models on the BCC surface defect database for detection performance. All experiments were conducted under the same parameter settings. The experimental results are shown in Table 8. Our experimental results on our custom dataset, using identical initial parameters as a baseline, clearly indicate that YOLOv5 showcased the most superior overall performance. This compelling evidence led us to select YOLOv5 as the reference model for our study. From Table 8, it can be observed that DCS-YOLO has the highest *mAP*_50_, P, and R, indicating its leading overall performance. Compared to the baseline model YOLOv5s, DCS-YOLO achieved a 5.1% increase in *mAP*_50_. Although the model size and computational complexity slightly increased, they are still within an acceptable range. Compared to the one-stage model SSD, DCS-YOLO achieved a 19.8% higher *mAP*_50_, a 3.9 faster FPS, and a reduction of 167.1 GFLOPs, with the model size approximately half of SSD. Compared to the two-stage model Faster R-CNN, DCS-YOLO achieved a 7.5% higher *mAP*_50_, a 123.7 faster FPS, a decrease of 178 GFLOPs, and the model size is only about one-third of Faster R-CNN.

**Table 8:** Comparative experiment.

Models	Backbone	Weights(M)	GFLOPs	FPS	P	R	*mAP*_50_(%)
SSD [38]	VGGNet-16	52.6	183.4	143.2	89.7	64.4	72.4
Faster R-CNN	ResNet50	83.0	194.3	23.4	65.8	84.9	84.7
YOLOv5n	Modified CSPv5	3.9	4.2	322.6	83.2	84.2	85.3
YOLOv5s		14.5	15.8	258.7	87.3	85.6	87.1
YOLOv5l		92.9	107.7	97.1	86.5	85.7	87.2
YOLOv5x		173.2	203.9	52.6	90.2	80.5	87.8
YOLOv5m		42.3	47.9	147.1	88.3	85.7	88.0
YOLOv7	YOLOv7	74.9	105.2	74.1	87.0	84.0	87.8
Mask R-CNN	Swin transformer	74.2	128.4	70.2	87.9	86.1	86.4
Hornet	Hornet	151.2	158.4	106.5	85.6	80.6	88.3
ConvNext	Convnext	139.4	154.4	148.4	87.7	87.1	88.4
YOLOv5	Mobilenetv3l	10.9	10.3	103.1	85.1	83.8	87.5
YOLOv5	Shufflenetv2	8.1	8.0	138.9	88.8	82.6	88.3
YOLOv5	Mobilenetv3s	41.2	38.3	138.9	89.3	85.7	88.8
YOLOv5	Mobileone	22.7	25.9	49.3	86.6	87.2	89.2
YOLOv9s	RepNCSPELAN4	20.3	38.7	97.1	85.2	86.9	89.6
LF-YOLO [39]	——	4.0	4.6	130.0	61.6	70.7	69.3
Next-ViT_Small [40]	Next-ViT_Small	68.0	95.8	32.6	63.5	65.9	68.6
DCS-YOLO-x	DCN-CoT3	297.3	206.8	32.2	86.1	86.9	89.2
DCS-YOLO-n		7.5	4.3	153.8	89.9	85.6	90.5
DCS-YOLO-l		163.9	109.6	54.3	86.8	88.5	90.6
DCS-YOLO-m		77.5	49.0	82	90.4	88.9	90.7
DCS-YOLO-s		28.1	16.2	147.1	90.7	90.2	92.2

**Fig 11:**
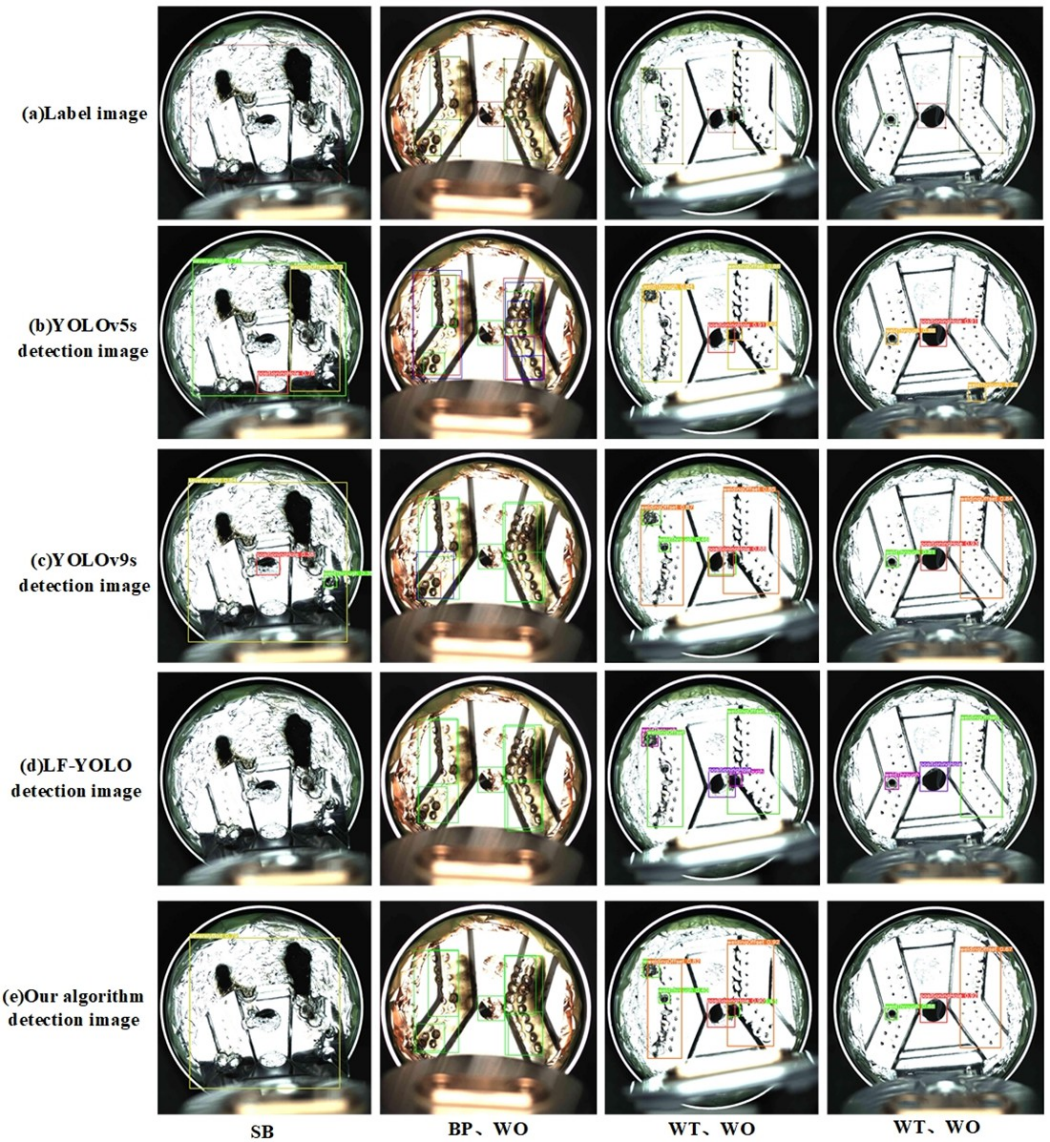
Detection effect image.

The Fig 11 demonstrates the detection performance of the baseline model and DCS-YOLO on the BCC surface defect database. Considering the small size and dense distribution of the *Bad point*, for comparison, we represent the samples with detected *Bad point* using red bounding boxes for false positives, blue bounding boxes for false negatives, and green bounding boxes for correct detections. We analyze from left to right. In the first column for the *Severely bad* category detection, the baseline model and YOLOv9s exhibited false positives, while LF-YOLO showed false negatives. Conversely, DCS-YOLO achieved high detection accuracy in accurately identifying defects. In the second column for *Bad point* and *Welding offset* category detection, the baseline model and YOLOv9s displayed varying degrees of false positives and false negatives. In the third column for *Welding through* and *Welding offset* category detection, the baseline model and LF-YOLO missed the small *Welding through* on the left, and YOLOv9s missed the *Welding through* on the right, whereas DCS-YOLO effectively addressed the issue of missing *Welding through* detections. In the fourth column for *Welding through* and *Welding offset* category detection, the baseline model missed *Welding offset* and also had false positives for *Welding through*. Overall, DCS-YOLO outperformed other models by effectively resolving issues of false positives and false negatives.

**Fig 12:**
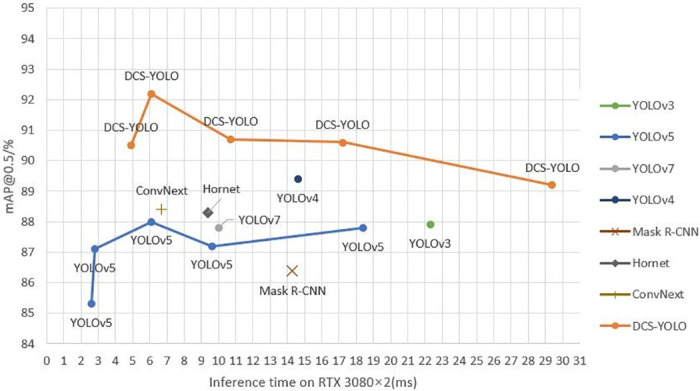
Graph of experimental results for different models.

The experimental results of several classical models are presented with line graphs, as shown in Fig 12. The horizontal axis represents model inference time, where smaller values indicate faster speeds. The vertical axis represents the average precision value of the models, where larger values indicate better performance. Models closer to the top-left corner of the graph demonstrate superior overall performance. From Fig 12, it can be observed that DCS-YOLO achieves an average inference time of 6.2ms, demonstrating the best speed.

#### Results of defect detection for different types

Experiment A, which involves multi-scale improvements on YOLOv5s, and experiment B, which builds upon A with DC enhancements (where *PH* stands for *Positioning hole*), are detailed. Table 9 shows the values of three metrics (*P*, *R*, *mAP*) for six target objects across YOLOv5s, A, and B. The table reveals that for the “Severely bad” defect category, experiment A’s detection results indicate a 21.5 increase in R and a 12.2% improvement in *mAP*_50_ compared to the baseline model. This demonstrates that the addition of detection layers effectively enhances the model’s ability to detect larger defects in the “*Severely bad*” category. Overall, while individual improvements did not achieve optimal results, the combination of all three enhancements resulted in the highest *mAP*_50_ across all six targets.

**Table 9:** Detection results of three indicators for six target objects on YOLOv5s, A, and B.

Indicators	P				R				*mAP*_50_(%)			
Defect	5s	A	B	ours	5s	A	B	ours	5s	A	B	ours
Test												
PH	97.3	97.4	96.9	**97.9**	**100**	**100**	**100**	100	97.9	98.6	98.3	**98.9**
BP	66.0	71.0	72.3	**76.3**	**67.2**	56.0	63.0	63.1	65.6	66.9	71.6	**75.3**
WO	93.5	91.8	93.0	**93.7**	96.3	96	**97.9**	97.5	97.2	97.2	96.8	**97.5**
NC	88.6	96.8	89.3	**97.6**	**100**	**100**	**100**	100	**99.5**	**99.5**	**99.5**	**99.5**
SB	**100**	**100**	**100**	**100**	71.6	93.1	84.6	**100**	87.3	99.5	99.5	**99.7**
WT	78.4	78.8	74.2	**78.9**	78.4	**81.1**	**81.1**	80.8	75.1	80.3	81.3	**82.8**
ALL	87.3	89.3	87.6	**90.7**	85.6	87.7	87.8	**90.2**	87.1	90.3	91.3	**92.2**

#### Generalization experiment

To assess the generalization capability of DCS-YOLO comprehensively, we conducted additional validation on the more challenging NEU-DET public dataset. As shown in Table 10, DCS-YOLO performed well on this dataset, achieving an mAP50 of 78.1% with a model size of only 28.1M. Its GFLOPs were slightly higher and FPS slightly lower compared to the baseline model. To ensure robust detection performance, the model size was slightly increased. However, all other metrics remained optimal. Experimental results demonstrate that DCS-YOLO exhibits strong detection performance, highlighting its robustness and effective generalization across diverse scenarios.

**Table 10:** Comparison of proposed model and existing models on NEU-DET.

Models	Backbone	Weights(M)	GFLOPs	FPS	P	R	*mAP*_50_(%)
YOLOv5s	Modified CSP v5	14.5	15.8	156.3	60.0	67.0	69.6
YOLOv8s	CSPDarknet-C2f	22.5	28.4	108.7	67.0	64.0	70.8
YOLOv9s	RepNCSPELAN4	20.3	38.7	96.2	73.0	69.6	75.6
DCS-YOLO	DCN-CoT3	28.1	16.2	133.3	73.5	75.2	78.1

## Conclusion

To address the surface defect detection in the battery current collector of electric vehicles, an improved target detection algorithm called DCS-YOLO based on YOLOv5 was proposed. In the model’s feature extraction phase, we enhance the multiscale capability and introduce additional detection layers to improve the learning capacity for extremely large object features. We incorporate the DC module into the backbone network to enlarge the receptive field, partially solving the problem of object fragmentation and better extracting adjacent key features to enhance feature extraction ability. Furthermore, we add SoftCBAM to the Neck to suppress irrelevant information and improve the model’s detection capability. Based on the experimental results, it can be observed that our proposed DCS-YOLO exhibits the best overall performance. Although DCS-YOLO slightly increases the size and computational complexity compared to the baseline model, it exhibits greater advantages in terms of detection accuracy. In our future work, we will continue to delve into researching and optimizing the structure of deep learning networks to achieve more efficient computation and superior performance. Our focus will be on simplifying and streamlining network architectures, utilizing advanced automated methods such as automatic network architecture search to discover and apply optimal designs. Concurrently, we aim to enhance the model’s ability to generalize across various datasets and tasks, ensuring robust and reliable performance in practical applications [41, 42].
